# Fault Diagnosis and Minimum Rational Entropy Fault Tolerant Control of Stochastic Distribution Collaborative Systems

**DOI:** 10.3390/e20110820

**Published:** 2018-10-24

**Authors:** Lina Yao, Wei Wu, Yunfeng Kang, Lifan Li

**Affiliations:** College of Electrical Engineering, Zhengzhou University, Zhengzhou 450001, China

**Keywords:** stochastic distribution collaborative control systems, fault diagnosis, fault tolerant control, minimum rational entropy control

## Abstract

In this paper, a fault-tolerant control scheme is presented for a class of stochastic distribution collaborative control systems, which are composed of three subsystems connected in series to complete the control target. The radial basis function neural network is used to approximate the output probability density function of the third subsystem, which is also the output of the entire system. When fault occurs in the first subsystem, an adaptive diagnostic observer is designed to estimate the value of fault. However, the first subsystem does not have the ability of self-recovery, minimum rational entropy controllers are designed in the latter subsystems to compensate the influence of the fault and minimize the entropy of the system output. A numerical simulation is given to verify the effectiveness of the proposed scheme.

## 1. Introduction

In recent years, fault diagnosis (FD) and fault tolerant control (FTC) of stochastic distribution systems have received significant attentions. A variety of FD and FTC techniques have been introduced in [1,2,3,4,5,6,7,8]. However, most of them aim at the single system. With the increasing complexity of modern engineering system, the majority of systems have multiple collaboration subsystems instead of a single system, which makes FD and FTC become more difficult. Different from the general system, the control object of the stochastic distribution control (SDC) systems is the probability density function (PDF) of the output rather than the output [2]. Thus, SDC systems no longer depend on the assumption that the variables of the system are subjected to Gaussian distribution, such as molecular weight distribution of chemical processes [9] and the distribution of the flame in the boiler [10].

In most systems, the noise is assumed to obey Gaussian distribution, which is not satisfied in the practical application. Therefore, entropy concept [11] is proposed to measure the uncertainty of the system output in SDC systems. In most of the existing FTC results, the target PDF is pre-specified. Thus, many tracking control methods can be applied directly. In [12,13], robust model predictive controller is presented for discrete nonlinear systems and constrained linear systems respectively. However, the expected output PDF is unknown in many cases. In response to this situation, the minimum entropy control algorithm can be applied to minimize the randomness or uncertainty of the system output. Recently, many achievements have been made in minimum entropy control [14,15]. In [16], the entropy has been used to characterize the uncertainty of the tracking error for general nonlinear and non-Gaussian stochastic systems. However, the fault tolerant control is not considered.

There are many ways to approximate the output PDF. B-spline function approximation is a popular method [6,7]. However, the parameter selection of radial basis function (RBF) is more flexible than B-spline model, which is used to approximate the output PDF in this paper. The form of RBF is usually used in most approximation processes [17,18]. A FD and model predictive control scheme for non-Gaussian stochastic distribution control systems based on T-S fuzzy model is proposed in [19], which the output PDF of SDC systems is approximated by RBF neural network.

Most of the existing literatures focus on the single SDC system and only a few research results focus on complex systems consisting of multiple subsystems. In [20], FD algorithm is proposed for the collaborative system composed of two SDC subsystems, iterative learning control is used to design the fault-tolerant controller so that the output PDF of the SDC systems can track the desired PDF. In [21,22], When fault occurs in subsystem 1 and subsystem 1 does not have the ability of self-recovery, a compensation item is added to the controller of subsystem 2, leading to fault tolerant control of the whole system. In above mentioned literatures the situation where the expected output PDF is unknown is not taken into account and the way of collaboration is similar.

The actual system is often composed of three or more subsystems. However, there is few research about the collaborative system consists of more than two subsystems, which will be studied in this paper. In order to be closer to reality, we study the collaborative system with three subsystems. The system parameters of the second subsystem are affected by the output PDF of the first system and the system parameters of the third subsystem are affected by the output PDF of the second system. Further, the situation where the expected output is unknown is considered. As the continuous definition of Shannon entropy may not meet the requirements (positive definite) of an index function [23], so we consider the new rational entropy performance index to carry out the controller design. When fault occurs in subsystem 1, the minimum rational entropy fault tolerant controller is designed in subsystem 2 and subsystem 3 respectively to minimize the uncertainty of the system output.

The rest of this paper is organized as follows. In Section 2, the system model is given. Section 3 presents a FD algorithm for the faulty subsystem using LMI techniques. A minimum entropy FTC controller is designed in Section 4. A simulation is given in Section 5.

## 2. Model Description

Denote the output y(t) of the SDC system as a uniformly bounded stochastic process defined on a known interval [a,b] at any sampling time *t*. Denote u(t) as the control input vector. Then the output y(t) can be represented by its probability density function γ(y,u(t)) as follows

$$P\left(a\leq y\left(t\right)<\tau |u\left(t\right)\right)=\int_{a}^{\tau }\gamma (y,u(t))dy$$

where P(a≤y(t)<τ|u(t)) is the probability and the output y(t) is within the interval [a,τ] under the action of u(t). It is assumed that the range of outputs [a,b] is known and the output PDF is measurable. The output PDF is approximated by predefined rational square-root radial basis functions which can be expressed as follows

(1) $$\begin{array}{c} \begin{array}{c} \sqrt{\gamma (y,u(t))} \end{array}=\frac{\sum_{i=1}^{n}w_{i}c_{i}\left(y\right)}{\sqrt{\sum_{i,j=1}^{n}w_{i}w_{j}\int_{a}^{b}c_{i}\left(y\right)c_{j}\left(y\right)dy}}=\frac{C(y)v(t)}{\sqrt{v^{T}\left(t\right)\Sigma v\left(t\right)}} \end{array}$$

where $C\left(y\right)=[c_{1}\left(y\right),c_{2}\left(y\right),\cdots ,c_{n}\left(y\right)]$ are chosen as *n* radial basis functions and $v\left(t\right)=[v_{1}\left(t\right),v_{2}\left(t\right),\cdots ,v_{n}\left(t\right)]$ are chosen as the corresponding weights, and $\Sigma =\int_{a}^{b}C^{T}\left(y\right)C\left(y\right)dy$.

The model of subsystem 1 can be described as follows

(2) $$\begin{array}{c} {\dot{x}}_{1}\left(t\right)=A_{1}x_{1}\left(t\right)+B_{1}u_{1}\left(t\right)+Hf\left(t\right)+Sd\left(t\right)\\ v_{1}\left(t\right)=D_{1}x_{1}\left(t\right)+E_{1}u_{1}\left(t\right)\\ \sqrt{\gamma_{1}\left(y,u_{1}\left(t\right)\right)}=\frac{C\left(y\right)v_{1}\left(t\right)}{\sqrt{v_{1}^{T}\left(t\right)\Sigma v_{1}\left(t\right)}} \end{array}$$

where $x_{1}\left(t\right)\in R^{l\times 1}$ is the system state vector, $u_{1}\left(t\right)\in R^{m\times 1}$ is the control input vector, $f\left(t\right)\in R^{r\times 1}$ is the fault vector, $d\left(t\right)\in R^{r\times 1}$ is the disturbance vector. When no fault occurs, f(t)=0. $A_{1},B_{1},D_{1},H,S$ are known constant matrices with appropriate dimensions, and $v_{1}\left(t\right)$ is the output weight vector.

The model of subsystem 2 can be expressed as

(3) $$\begin{array}{c} {\dot{x}}_{2}\left(t\right)=A_{2}\left(v_{1}\left(t\right)\right)x_{2}\left(t\right)+B_{2}\left(v_{1}\left(t\right)\right)u_{2}\left(t\right)+Sd\left(t\right)\\ v_{2}\left(t\right)=D_{2}\left(v_{1}\left(t\right)\right)x_{2}\left(t\right)+E_{2}\left(v_{1}\left(t\right)\right)u_{2}\left(t\right)\\ \sqrt{\gamma_{2}\left(y,u_{2}\left(t\right)\right)}=\frac{C\left(y\right)v_{2}\left(t\right)}{\sqrt{v_{2}^{T}\left(t\right)\Sigma v_{2}\left(t\right)}} \end{array}$$

where $x_{2}\left(t\right)\in R^{m\times 1}$ is the system state vector, $u_{2}\left(t\right)\in R^{m\times 1}$ is the control input vector, $v_{2}\left(t\right)$ is the output weight vector, $A_{2}\left(v_{1}\left(t\right)\right),B_{2}\left(v_{1}\left(t\right)\right)$, $D_{2}\left(v_{1}\left(t\right)\right)$ and $E_{2}\left(v_{1}\left(t\right)\right)$ ($A_{2},B_{2},D_{2},E_{2}$ for short) are parameter matrices affected by the weights of subsystem 1. Thus, $A_{2},B_{2},D_{2},E_{2}$ are time-varying matrices when the weights of subsystem 1 change.

The model of subsystem 3 can be expressed as

(4) $$\begin{array}{c} {\dot{x}}_{3}\left(t\right)=A_{3}\left(v_{2}\left(t\right)\right)x_{3}\left(t\right)+B_{3}\left(v_{2}\left(t\right)\right)u_{3}\left(t\right)+Sd\left(t\right)\\ v_{3}\left(t\right)=D_{3}\left(v_{2}\left(t\right)\right)x_{3}\left(t\right)+E_{3}\left(v_{2}\left(t\right)\right)u_{3}\left(t\right)\\ \sqrt{\gamma_{3}\left(y,u_{3}\left(t\right)\right)}=\frac{C\left(y\right)v_{3}\left(t\right)}{\sqrt{v_{3}^{T}\left(t\right)\Sigma v_{3}\left(t\right)}} \end{array}$$

where $x_{3}\left(t\right)\in R^{m\times 1}$ is the system state vector, $u_{3}\left(t\right)\in R^{m\times 1}$ is the control input vector, $v_{3}\left(t\right)$ is the output weight vector, $A_{3}\left(v_{2}\left(t\right)\right),B_{3}\left(v_{2}\left(t\right)\right)$, $D_{3}\left(v_{2}\left(t\right)\right)$ and $E_{3}\left(v_{2}\left(t\right)\right)$ ($A_{3},B_{3},D_{3},E_{3}$ for short) are parameter matrices affected by the weights of subsystem 2. Thus, $A_{3},B_{3},D_{3},E_{3}$ are time-varying matrices when the weights of subsystem 2 change.

**Figure 1:**
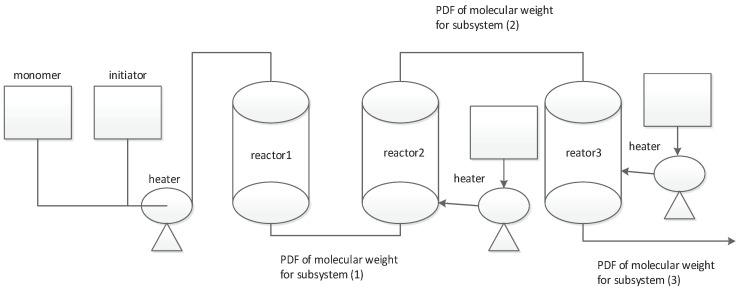
Fault and fault estimation of subsystem 1.

It can be seen from the state equations of three subsystems that the system parameters of subsystem 2 are affected by subsystem 1, and the system parameters of subsystem 3 are affected by subsystem 2. A typical case is molecular weight control in chemical reactions. As shown in Figure 1, during the chemical reaction, the desired product is often obtained by multi-step chemical reactions. The monomer and the initiator are reacted in the first reactor and then sent to the second chemical reactor. The other initiator is added for the second chemical reaction and the product is sent to the third reactor. Finally, after multi-step reaction to obtain the desired product, the output of the third reactor is the output of the entire system.

## 3. Fault Diagnosis

When fault occurs in subsystem 1, a FD algorithm is presented to estimate the size of the fault. An adaptive fault diagnosis observer is constructed as follows

(5) $$\begin{array}{c} {\dot{\hat{x}}}_{1}\left(t\right)=A_{1}{\hat{x}}_{1}\left(t\right)+B_{1}u_{1}\left(t\right)+H\hat{f}\left(t\right)+L\varepsilon \left(t\right)\\ {\hat{v}}_{1}\left(t\right)=D_{1}{\hat{x}}_{1}\left(t\right)+E_{1}u_{1}\left(t\right)\\ \sqrt{{\hat{\gamma }}_{1}\left(y,u_{1}\left(t\right)\right)}=\frac{C\left(y\right){\hat{v}}_{1}\left(t\right)}{\sqrt{{\hat{v}}_{1}^{T}\left(t\right)\Sigma {\hat{v}}_{1}\left(t\right)}}\\ \varepsilon \left(t\right)=\int_{a}^{b}\sigma \left(y\right)\left(\sqrt{\gamma_{1}\left(y,u_{1}\left(t\right)\right)}-\sqrt{{\hat{\gamma }}_{1}\left(y,u_{1}\left(t\right)\right)}\right)dy \end{array}$$

where ${\hat{x}}_{1}\left(t\right),{\hat{v}}_{1}\left(t\right)$ and $\hat{f}\left(t\right)$ are the estimation of state, weight and fault vector, respectively. ε(t) is the residual signal, *L* is the gain vector, which will be defined later, σ(y) is a pre-specified weighting function.

The residual signal can be obtained as

(6) $$\begin{array}{c} \varepsilon \left(t\right)=\int_{a}^{b}\sigma \left(y\right)\left(\sqrt{\gamma_{1}\left(y,u_{1}\left(t\right)\right)}-\sqrt{{\hat{\gamma }}_{1}\left(y,u_{1}\left(t\right)\right)}\right)dy\\ =\Lambda (\frac{v_{1}\left(t\right)}{\sqrt{v_{1}^{T}\left(t\right)\Sigma v_{1}\left(t\right)}}-\frac{{\hat{v}}_{1}\left(t\right)}{\sqrt{{\hat{v}}_{1}^{T}\left(t\right)\Sigma {\hat{v}}_{1}\left(t\right)}})\\ =\frac{\Lambda D_{1}e_{1}}{\sqrt{{\hat{v}}_{1}^{T}\left(t\right)\Sigma {\hat{v}}_{1}\left(t\right)}}-\frac{\Lambda v_{1}\left(t\right)}{\sqrt{{\hat{v}}_{1}^{T}\left(t\right)\Sigma {\hat{v}}_{1}\left(t\right)}}+\frac{\Lambda v_{1}\left(t\right)}{\sqrt{v_{1}^{T}\left(t\right)\Sigma v_{1}\left(t\right)}} \end{array}$$

where $\Lambda =\int_{a}^{b}\sigma (y)C(y)dy$.

Denote

$$\begin{array}{c} e_{1}\left(t\right)=x_{1}\left(t\right)-{\hat{x}}_{1}\left(t\right)\\ \tilde{f}\left(t\right)=f\left(t\right)-\hat{f}\left(t\right) \end{array}$$

**Assumption** **1.** *Suppose that $∥f∥\leq \frac{\alpha }{2}\;$, where α is a positive constant.*

**Assumption** **2.** *Suppose that $∥d∥\leq \theta \;$, where θ is a positive constant.*

**Lemma** **1** **[24].** *There exists $\lambda (T_{1}\leq \left|\lambda \right|\leq T_{2},\;T_{1}=\lambda_{\min}(\Sigma )/\sqrt{\lambda_{\max}(\Sigma )},\;T_{2}=\lambda_{\max}(\Sigma )/\sqrt{\lambda_{\min}(\Sigma )})$, such that the following inequality holds*

(7) $$\begin{array}{c} \sqrt{v_{1}^{T}\Sigma v_{1}}-\sqrt{{\hat{v}}_{1}^{T}\Sigma {\hat{v}}_{1}}=\lambda (\sqrt{v_{1}^{T}v_{1}}-\sqrt{{\hat{v}}_{1}^{T}{\hat{v}}_{1}}) \end{array}$$

Define $L=K\sqrt{{\hat{v}}^{T}\Sigma \hat{v}}$, and *K* is chosen to make the matrix $A_{1}-K\Lambda D_{1}$ be a Hurwitz matrix, *L* is the time-varying observer gain vector. The observed error dynamic system can be obtained as follows

(8) $$\begin{array}{c} {\dot{e}}_{1}\left(t\right)=A_{1}e_{1}\left(t\right)+H\tilde{f}\left(t\right)-L\varepsilon \left(t\right)+Sd\left(t\right)\\ =A_{1}e_{1}\left(t\right)+H\tilde{f}\left(t\right)-L\left(\frac{\Lambda D_{1}e_{1}}{\sqrt{{\hat{v}}_{1}^{T}\left(t\right)\Sigma {\hat{v}}_{1}\left(t\right)}}-\frac{\Lambda v_{1}\left(t\right)}{\sqrt{{\hat{v}}_{1}^{T}\left(t\right)\Sigma {\hat{v}}_{1}\left(t\right)}}+\frac{\Lambda v_{1}\left(t\right)}{\sqrt{v_{1}^{T}\left(t\right)\Sigma v_{1}\left(t\right)}}\right)+Sd\left(t\right)\\ =\left(A_{1}-K\Lambda D_{1}\right)e_{1}+H\tilde{f}\left(t\right)-K\Lambda v_{1}\left(t\right)\frac{\lambda (∥v_{1}\left(t\right)∥-∥{\hat{v}}_{1}\left(t\right)∥)}{\sqrt{v_{1}^{T}\left(t\right)\Sigma v_{1}\left(t\right)}}+Sd\left(t\right) \end{array}$$

The adaptive tuning law of $\hat{f}\left(t\right)$ is designed as follows

(9) $$\begin{array}{c} \dot{\hat{f}}\left(t\right)=-\Gamma \sqrt{{\hat{v}}_{1}^{T}\left(t\right)\Sigma {\hat{v}}_{1}\left(t\right)}\varepsilon \left(t\right) \end{array}$$

**Theorem** **1.** *If there exist* Γ *and two positive definite symmetric matrices P and Q such that the following condition is satisfied*

(10) $$\begin{array}{c} (A_{1}-K\Lambda D_{1}{)}^{T}P+P(A_{1}-K\Lambda D_{1})=-Q \end{array}$$

*then the observation error system is stable.*

**Proof.**  A Lyapunov function is selected as follows:

(11) $$\begin{array}{c} \Pi =e_{1}^{T}Pe_{1}+{\tilde{f}}^{T}\tilde{f} \end{array}$$

Then the first-order derivative can be obtained as follows

(12) $$\begin{array}{c} \dot{\Pi }=e_{1}^{T}\left[{\left(A_{1}-K\Lambda D_{1}\right)}^{T}P+P\left(A_{1}-K\Lambda D_{1}\right)\right]e_{1}+2e_{1}^{T}PH\tilde{f}\\ -2e_{1}^{T}PK\Lambda v_{1}\left(t\right)\frac{\lambda ∥v_{1}\left(t\right)∥-∥{\hat{v}}_{1}\left(t\right)∥}{\sqrt{v_{1}^{T}\left(t\right)\Sigma v_{1}\left(t\right)}}+2e_{1}^{T}PSd_{1}\left(t\right)\\ -2{\tilde{f}}^{T}\Gamma \Lambda D_{1}e_{1}-2{\tilde{f}}^{T}\Gamma \Lambda v_{1}\left(t\right)\frac{\lambda (∥v_{1}\left(t\right)∥-∥{\hat{v}}_{1}\left(t\right)∥)}{\sqrt{v_{1}^{T}\left(t\right)\Sigma v_{1}\left(t\right)}}\\ \leq -\left(\lambda_{\min}\left(Q\right)+2\frac{T_{2}∥PK\Lambda D∥}{\sqrt{∥\Sigma ∥}}\right){∥e_{1}∥}^{2}+∥e_{1}∥(∥PH-D_{1}^{T}\Lambda^{T}\Gamma ∥\\ -\frac{T_{2}∥\Gamma \Lambda D∥}{\sqrt{∥\Sigma ∥}})∥\tilde{f}∥+2∥e_{1}∥∥PS∥∥d_{1}∥ \end{array}$$

Denote

$$\begin{array}{c} M_{1}=\lambda_{\min}(Q)+2\frac{T_{2}∥PK\Lambda D∥}{\sqrt{∥\Sigma ∥}},M_{2}=∥PH-D_{1}^{T}\Lambda^{T}\Gamma ∥-\frac{T_{2}∥\Gamma \Lambda D∥}{\sqrt{∥\Sigma ∥}},M_{3}=2∥PS∥ \end{array}$$

Then

$$\dot{\Pi }\leq -M_{1}{∥e_{1}∥}^{2}+∥e_{1}∥\left(M_{2}\alpha +M_{3}\theta \right)=-M_{1}{\left(∥e_{1}∥-\frac{\left(M_{2}\alpha +M_{3}\theta \right)}{2M_{1}}\right)}^{2}+\frac{{\left(M_{2}\alpha +M_{3}\theta \right)}^{2}}{4M_{1}}$$

Therefore when $∥e_{1}∥\geq \frac{M_{2}\alpha +M_{3}\theta }{M_{1}}$ holds, it can be obtained that $\dot{\Pi }\leq 0$. The dynamic observation error system (8) is stable. ☐

## 4. Fault Tolerant Control

In this section, the desired PDF is unknown. Minimum rational entropy controllers are designed in the second and third subsystem respectively.

In subsystem 2, the performance function is selected as follows:

(13) $$\begin{array}{c} J=-\int_{a}^{b}\gamma_{2}\left(y,u_{2}\left(t\right)\right)\ln\frac{\gamma_{2}\left(y,u_{2}\left(t\right)\right)}{1+\gamma_{2}\left(y,u_{2}\left(t\right)\right)}dy+{\left(\mu_{2}-\mu_{g}\right)}^{2}+u_{2}^{T}\left(t\right)Ru_{2}\left(t\right)\; \end{array}$$

where the first term is the rational entropy of the output variables, the rational entropy reflects the uncertainty of the system. The second term is the error between the mean $\mu_{2}=\int_{a}^{b}y\gamma_{2}(y,u_{2}(t))dy$, and target mean $\mu_{g}$. The third term is a natural quadratic constraint for the control input, where $R=R^{T}>0$. The performance index has certain limitations, mainly because the entropy is a concave function, and the minimum value is more than one. This in turn leads to a design controller that cannot predict where it will be stable, or that the target is unpredictable. It is well known that the mean value indicates the center position of the random variable, so it seems more reasonable at a certain central position.

The purpose of designing the minimum rational entropy controller is to find the required optimal control input u(t) to minimize the performance function.

To simplify the calculation, the performance function (13) is divided into two parts

(14) $$\begin{array}{c} J_{1}=-\int_{a}^{b}\gamma_{2}(y,u_{2}(t))\ln\frac{\gamma_{2}(y,u_{2}(t))}{1+\gamma_{2}(y,u_{2}(t))}dy,J_{2}=(\mu_{2}-\mu_{g}{)}^{2} \end{array}$$

It is known that

$$\gamma_{2}(y,u_{2}(t))=\frac{{(C(y)v_{2}(t))}^{T}C(y)v_{2}(t)}{v_{2}^{T}(t)\Sigma v_{2}(t)}$$

Denote

$$\begin{array}{c} N=\frac{\partial \gamma_{2}(y,u_{2}(t))}{\partial v_{2}(t)}\\ =\frac{2(v_{2}^{T}(t)C^{T}(y))(C(y)(v_{2}^{T}(t)\Sigma v_{2}(t))-(C(y)v_{2}(t))(v_{2}^{T}(t)\Sigma ))}{{(v_{2}^{T}(t)\Sigma v_{2}(t))}^{2}} \end{array}$$

From Equation (14), the derivative of $J_{1}$ and $J_{2}$ can be obtained as follows

$$\begin{array}{c} \frac{\partial J_{1}}{\partial u_{2}}=-\int_{a}^{b}(\frac{\partial \gamma_{2}(y,u_{2}(t))}{\partial v_{2}(t)}\frac{\partial v_{2}(t)}{\partial u_{2}(t)}\ln\frac{\gamma_{2}(y,u_{2}(t))}{1+\gamma_{2}(y,u_{2}(t))}+\frac{1}{1+\gamma_{2}(y,u_{2}(t))}\frac{\partial \gamma_{2}(y,u_{2}(t))}{\partial v_{2}(t)}\frac{\partial v_{2}(t)}{\partial u_{2}(t)})dy\\ =-\int_{a}^{b}\left[\ln\frac{\gamma_{2}(y,u_{2}(t))}{1+\gamma_{2}(y,u_{2}(t))}+\frac{1}{1+\gamma_{2}(y,u_{2}(t))}\right]Ndy\frac{\partial v_{2}(t)}{\partial u_{2}(t)} \end{array}$$

$$\frac{\partial J_{2}}{\partial u_{2}}=2(\mu_{2}-\mu_{g})\int_{a}^{b}yNdy\frac{\partial v_{2}(t)}{\partial u_{2}(t)}$$

From (3), it can be calculated that

$$\frac{\partial v_{2}(t)}{\partial u_{2}(t)}=E_{2}$$

Combining the above equations, the derivative of the performance function can be obtained as follows

$$\begin{array}{c} \frac{\partial J}{\partial u_{2}}=-\int_{a}^{b}\left[\ln\frac{\gamma_{2}(y,u_{2}(t))}{1+\gamma_{2}(y,u_{2}(t))}+\frac{1}{1+\gamma_{2}(y,u_{2}(t))}\right]NdyE_{2}+2(\mu_{2}-\mu_{g})\int_{a}^{b}yNdyE_{2}+2Ru_{2}(t) \end{array}$$

The optimal controller of subsystem 2 can be obtained by solving $\frac{\partial J}{\partial u_{2}(t)}=0$

(15) $$\begin{array}{c} u_{2}(t)=\frac{1}{2R}\left\{\int_{a}^{b}\left[\ln\frac{\gamma_{2}(y,u_{2}(t))}{1+\gamma_{2}(y,u_{2}(t))}+\frac{1}{1+\gamma_{2}(y,u_{2}(t))}\right]NdyE_{2}-2(\mu_{2}-\mu_{g})\int_{a}^{b}yNdyE_{2}\right\} \end{array}$$

In a similar way, in subsystem 3, the performance function is selected as follows

(16) $$\begin{array}{c} Z=-\int_{a}^{b}\gamma_{3}\left(y,u_{3}\left(t\right)\right)\ln\frac{\gamma_{3}\left(y,u_{3}\left(t\right)\right)}{1+\gamma_{3}\left(y,u_{3}\left(t\right)\right)}dy+{\left(\mu_{3}-\mu_{g}\right)}^{2}+u_{3}^{T}\left(t\right)Ru_{3}\left(t\right) \end{array}$$

where the first term is the entropy of the output variable, the second term is the error between the mean $\mu_{3}=\int_{a}^{b}y\gamma_{3}(y,u_{3}(t))dy$, and target mean $\mu_{g}$, and the third term is a natural quadratic constraint for the control input, where $R=R^{T}>0$.

To simplify the calculation, the performance function (16) is divided into two parts

(17) $$\begin{array}{c} Z_{1}=-\int_{a}^{b}\gamma_{3}(y,u_{3}(t))\ln\frac{\gamma_{3}(y,u_{3}(t))}{1+\gamma_{3}(y,u_{3}(t))}dy,Z_{2}=(\mu_{3}-\mu_{g}{)}^{2} \end{array}$$

It is known that

$$\gamma_{3}(y,u_{3}(t))=\frac{{(C(y)v_{3}(t))}^{T}C(y)v_{3}(t)}{v_{3}^{T}(t)\Sigma v_{3}(t)}$$

Denote

$$\begin{array}{c} M=\frac{\partial \gamma_{3}(y,u_{3}(t))}{\partial v_{3}(t)}\\ =\frac{2(v_{3}^{T}(t)C^{T}(y))(C(y)(v_{3}^{T}(t)\Sigma v_{3}(t))-(C(y)v_{3}(t))(v_{3}^{T}(t)\Sigma ))}{{(v_{3}^{T}(t)\Sigma v_{3}(t))}^{2}} \end{array}$$

The Equation (17) can be further formulated as follows

$$\begin{array}{c} \frac{\partial Z_{1}}{\partial u_{3}}=-\int_{a}^{b}(\frac{\partial \gamma_{3}(y,u_{3}(t))}{\partial v_{3}(t)}\frac{\partial v_{3}(t)}{\partial u_{3}(t)}\ln\frac{\gamma_{3}(y,u_{3}(t))}{1+\gamma_{3}(y,u_{3}(t))}+\frac{1}{1+\gamma_{3}(y,u_{3}(t))}\frac{\partial \gamma_{3}(y,u_{3}(t))}{\partial v_{3}(t)}\frac{\partial v_{3}(t)}{\partial u_{3}(t)})dy\\ =-\int_{a}^{b}\left[\ln\frac{\gamma_{3}(y,u_{3}(t))}{1+\gamma_{3}(y,u_{3}(t))}+\frac{1}{1+\gamma_{3}(y,u_{3}(t))}\right]Mdy\frac{\partial v_{3}(t)}{\partial u_{3}(t)} \end{array}$$

$$\frac{\partial Z_{2}}{\partial u_{3}}=2(\mu_{3}-\mu_{g})\int_{a}^{b}yMdy\frac{\partial v_{3}(t)}{\partial u_{3}(t)}$$

From (4), it can be calculated that

$$\frac{\partial v_{3}(t)}{\partial u_{3}(t)}=E_{3}$$

The derivative of the performance function can be obtained as follows

$$\begin{array}{c} \frac{\partial Z}{\partial u_{3}}=-\int_{a}^{b}\left[\ln\frac{\gamma_{3}(y,u_{3}(t))}{1+\gamma_{3}(y,u_{3}(t))}+\frac{1}{1+\gamma_{3}(y,u_{3}(t))}\right]MdyE_{3}+2(\mu_{3}-\mu_{g})\int_{a}^{b}yMdyE_{3}+2Ru_{3}(t) \end{array}$$

The optimal controller of subsystem 2 can be obtained by solving $\frac{\partial Z}{\partial u_{3}(t)}=0$

(18) $$\begin{array}{c} u_{3}(t)=\frac{1}{2R}\left\{\int_{a}^{b}\left[\ln\frac{\gamma_{3}(y,u_{3}(t))}{1+\gamma_{3}(y,u_{3}(t))}+\frac{1}{1+\gamma_{3}(y,u_{3}(t))}\right]MdyE_{3}-2(\mu_{3}-\mu_{g})\int_{a}^{b}yMdyE_{3}\right\} \end{array}$$

To compensate the fault occurred in subsystem 1, the actual controller is as follows

(19) $$\begin{array}{c} u_{3}(t)=\frac{1}{2R}(\int_{a}^{b}\left[\ln\frac{\gamma_{3}(y,u_{3}(t))}{1+\gamma_{3}(y,u_{3}(t))}+\frac{1}{1+\gamma_{3}(y,u_{3}(t))}\right]Mdy-2(\mu_{3}-\mu_{g})\int_{a}^{b}yMdy)(E_{3}+H\hat{f}) \end{array}$$

When the expected output PDF is not known in advance, the minimum entropy control is usually used to minimum the output uncertainty of the system. For the Shannon entropy performance index, it may be possible to make the non-negative property of the PDF not be satisfied. There is no such weakness for the rational entropy performance index.

## 5. A Simulation Example

An example of molecular weight control in chemical reactions in Figure 1 is used to prove the validity of the proposed algorithm. The mathematical model of the first reactor is given as follows

$$\begin{array}{c} {\dot{I}}_{1}\left(t\right)=\frac{I_{0}\left(t\right)-I_{1}\left(t\right)}{\theta }-K_{d}I_{1}\left(t\right)+K_{p}\sin\left(t\right)+K_{I1}u_{11}\left(t\right)\\ {\dot{M}}_{1}\left(t\right)=\frac{M_{0}\left(t\right)-M_{1}\left(t\right)}{\theta }-2K_{i}I_{1}\left(t\right)+K_{p}\sin\left(t\right)+K_{I2}u_{11}\left(t\right)-\left(K_{r}+K_{trm}\right)M_{1}\left(t\right)R_{i} \end{array}$$

where $I_{0}$ is the initial concentration of initiator ($\mathrm{mol}\cdot mL^{-1}$); $I_{1}$ is the initiator concentration (mol·mL${}^{-1}$); $\theta =\frac{V_{\theta }}{F_{\theta }}$ is the average residence time of the reactants in the reactor (s), $V_{\theta }$ is the volume of the reactor (mL), $F_{\theta }$ is the inlet material flow (mL·s${}^{-1}$); $M_{0}$ is the initial concentration of monomer (mol·mL${}^{-1}$); $M_{1}$ is the monomer concentration (mol·mL${}^{-1}$); $K_{d},K_{i},K_{r},K_{trm}$ are the reaction rate constants; $K_{I1}$ and $K_{I2}$ are the constants related to the control input; $K_{p}$ is the disturbance parameter; $u_{11}$ is the control input which is defined as $u_{11}=\frac{F_{M}}{F_{M}+F_{I}}$, where $F_{M}$ is the flow of monomer (mL·s${}^{-1}$) and $F_{I}$ is the flow of initiator (mL·s${}^{-1}$). $R_{i}$ is the concentration of the free radical. When the reaction in the first reactor is completed, it will be further reacted in the second reactor. The output of the first reactor will affect the system state of the second reactor, It is same in the third reactor.

Model parameter matrices are as follows

$$A_{1}=\left[\begin{array}{cc} -0.5 & 0\\ -0.5 & -1 \end{array}\right],B_{1}=\left[\begin{array}{c} 0.2\\ 0.3 \end{array}\right],H=\left[\begin{array}{c} 1\\ 2 \end{array}\right],D_{1}=\left[\begin{array}{cc} 1 & 0\\ 0 & 1 \end{array}\right],E_{1}=S=\left[\begin{array}{c} 1\\ 1 \end{array}\right]$$

$$A_{2}\left(v_{1}\left(t\right)\right)=\left[\begin{array}{cc} -0.5v_{11}\left(t\right) & 0\\ 1 & -0.2v_{12}\left(t\right) \end{array}\right],B_{2}\left(v_{1}\left(t\right)\right)=\left[\begin{array}{c} -0.7v_{11}\left(t\right)\\ 0.1v_{12}\left(t\right) \end{array}\right],E_{2}\left(v_{1}\left(t\right)\right)=\left[\begin{array}{c} 1.6v_{11}\left(t\right)\\ 2v_{12}\left(t\right) \end{array}\right]$$

$$\begin{array}{c} A_{3}\left(v_{2}\left(t\right)\right)=\left[\begin{array}{cc} -0.7v_{21}\left(t\right) & 0\\ 1 & -0.2v_{22}\left(t\right) \end{array}\right],B_{3}\left(v_{2}\left(t\right)\right)=\left[\begin{array}{c} 1.4v_{21}\left(t\right)\\ 0.9v_{22}\left(t\right) \end{array}\right],\\ D_{3}=D_{2}=D_{1},E_{3}\left(v_{2}\left(t\right)\right)=\left[\begin{array}{c} v_{21}\left(t\right)\\ v_{22}\left(t\right) \end{array}\right],d\left(t\right)=0.001*\sin\left(t\right) \end{array}$$

Denoting y∈[2,7), three radial basis functions are as follows

$$\begin{array}{c} c_{1}\left(y\right)=\exp\left(-{\left(y-3.5\right)}^{2}/1.5\right)\\ c_{2}\left(y\right)=\exp\left(-{\left(y-5.5\right)}^{2}/1.5\right) \end{array}$$

where the center vector is chosen as μ=[3.55.5] and the width is chosen as σ=1.5. The sampling time is assumed as 0.1s and the total simulation time is supposed as 100s.

To validate the algorithm, it is assumed that the fault has the following form

$$f\left(t\right)=\left\{\begin{array}{c} 0\;\;t\leq 50\\ 0.5\;t>50 \end{array}\right.$$

The gain of the observer and the FD learning law is chosen as follows

$$K=\left[\begin{array}{c} 0.26\\ \begin{array}{c} 0.36 \end{array} \end{array}\right],\Gamma =-1.93$$

**Figure 2:**
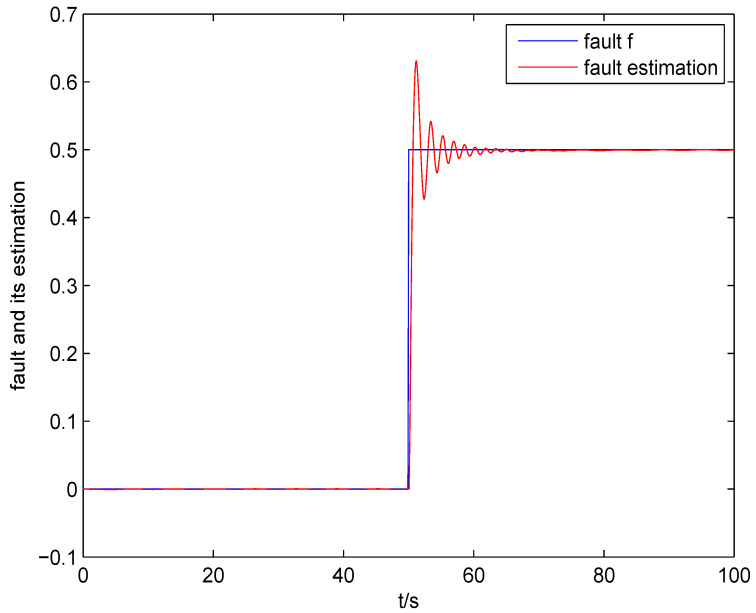
Fault and fault estimation of subsystem 1.

**Figure 3:**
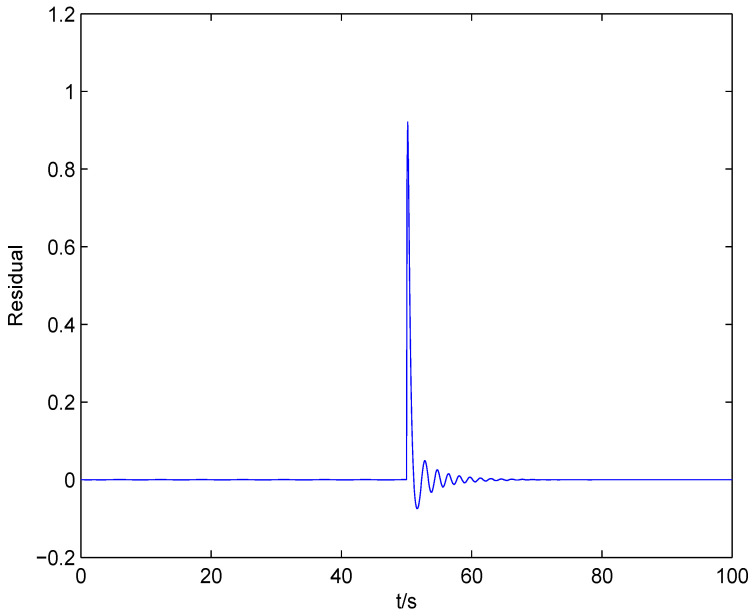
The response of the residual of subsystem 1.

**Figure 4:**
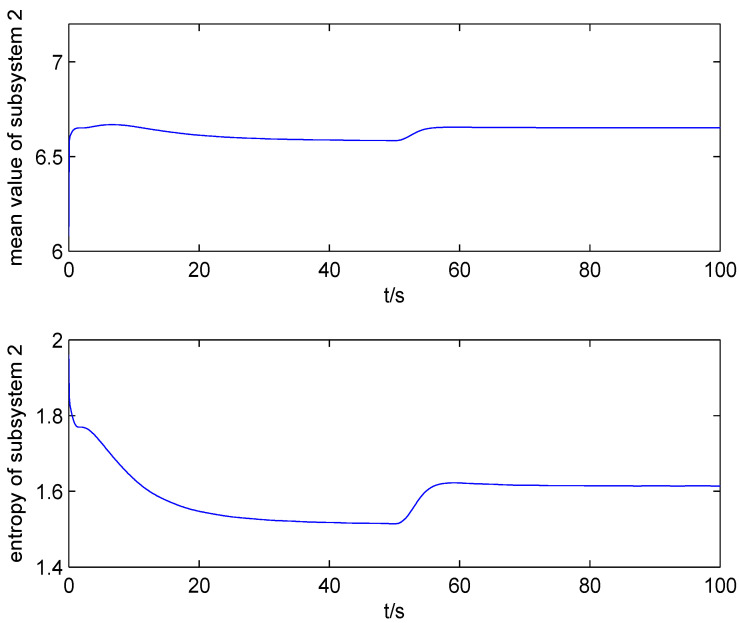
The mean value and rational entropy of subsystem 2.

**Figure 5:**
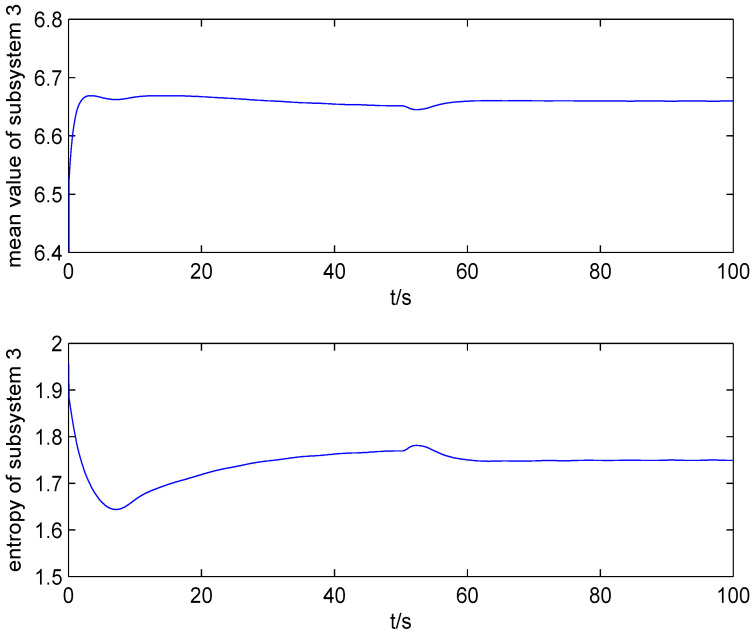
The mean value and rational entropy of subsystem 3.

**Figure 6:**
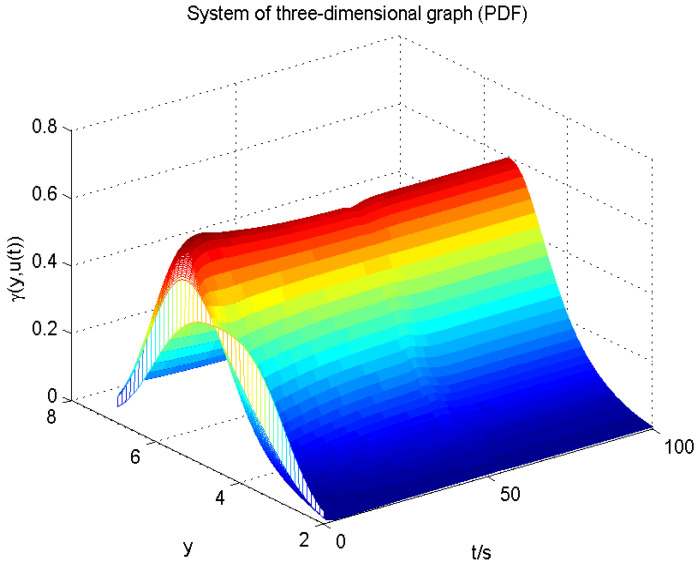
The output probability density function (PDF) of subsystem 3 with the rational Entropy fault tolerant control.

**Figure 7:**
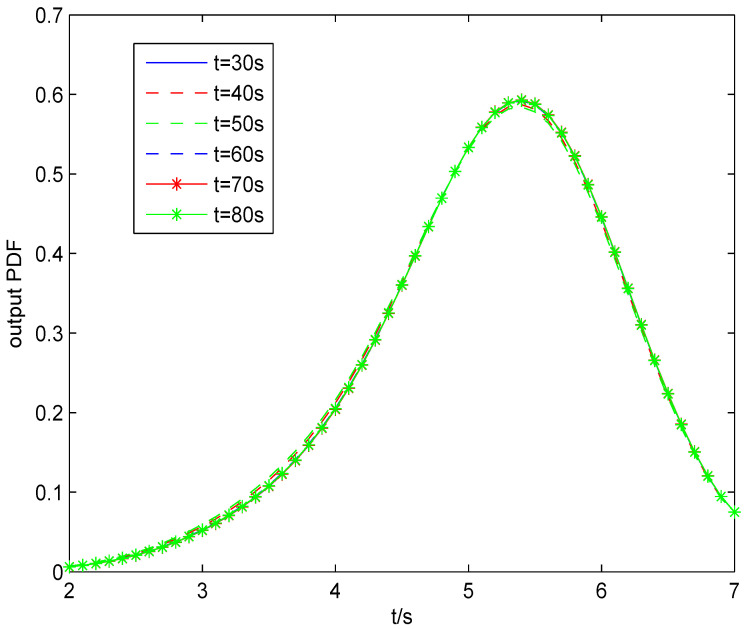
The output PDF with the rational Entropy fault tolerant control from 30s to 80s.

The result of fault estimation is presented in Figure 2 and Figure 3. It can be seen from Figure 2 that the fault diagnosis observer can quickly estimate the value of fault when fault occurs in subsystem 1. The fault estimation error in Figure 3 is small. The mean value and rational entropy of subsystem 2 are presented in Figure 4. Figure 5 shows the mean and rational entropy of subsystem 3. Fault occurs in subsystem 1 at 50s, and the rational entropy is affected by the fault. Then rational entropy decreases under the action of the fault tolerant controller. The output PDF of subsystem 3 is shown in Figure 6. Figure 7 shows the PDF by a 2D plot. It can be seen that the PDF after the fault tolerance is the same as the PDF before the fault occurred and the control input drives the system towards the direction of less randomness.

**Figure 8:**
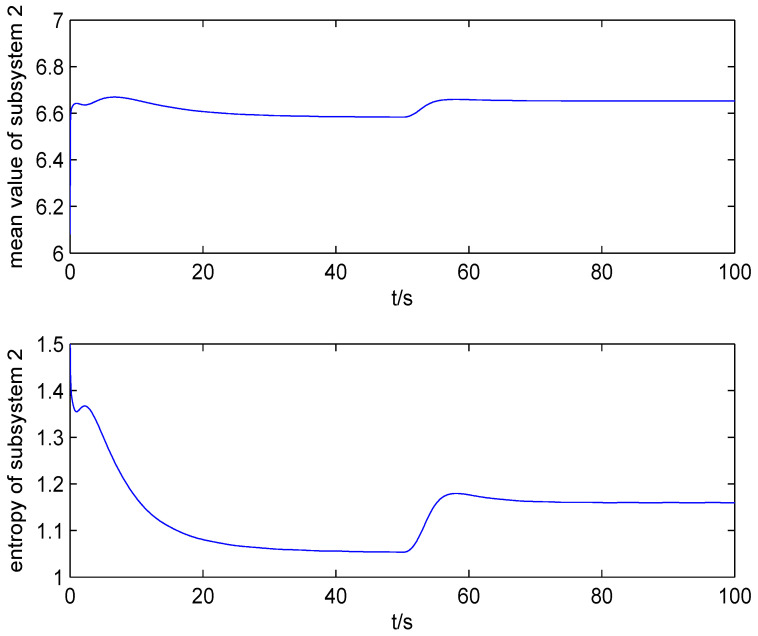
The mean value and Shannon entropy of subsystem 2.

**Figure 9:**
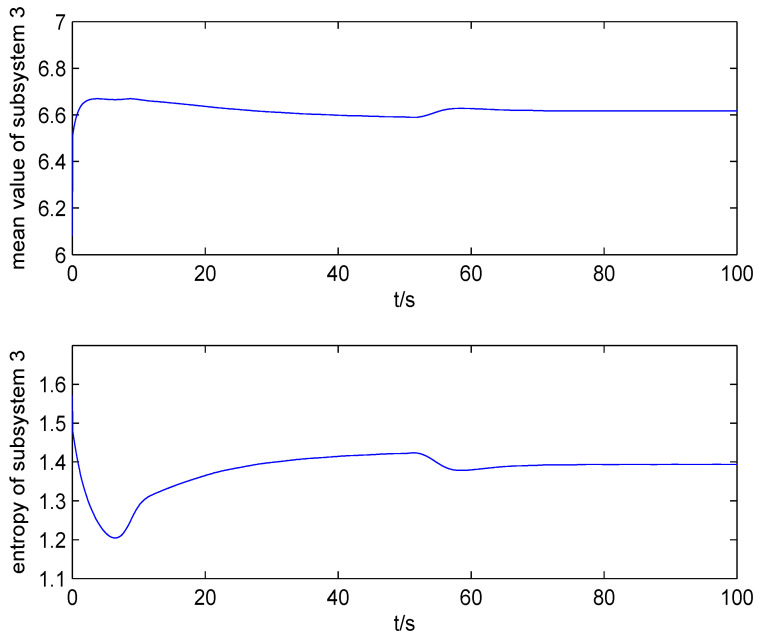
The mean value and Shannon entropy of subsystem 3.

**Figure 10:**
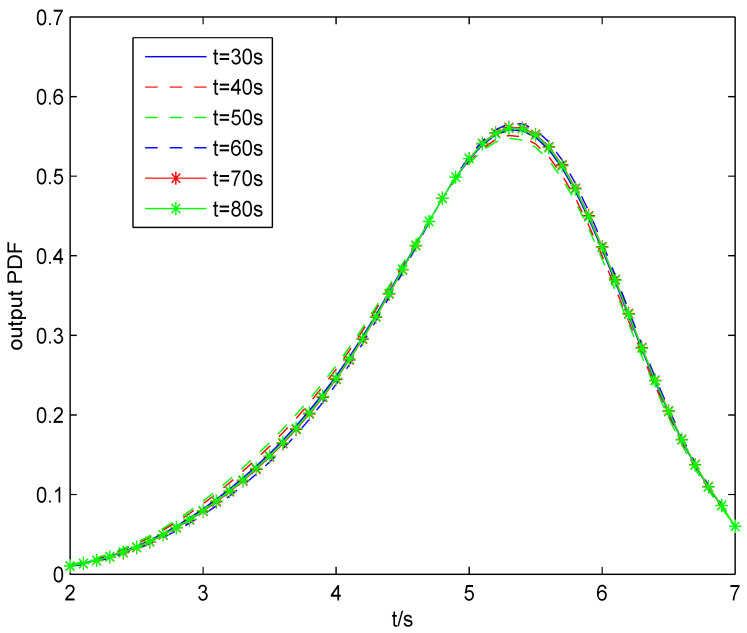
The output PDF with the Shannon Entropy fault tolerant control from 30s to 80s.

Figure 8 and Figure 9 show the mean value and Shannon entropy of subsystem 2 and subsystem 3 with the minimum Shannon entropy fault tolerant controller. The value of Shannon entropy is lower than that of the rational entropy because the type of two entropies is different. Figure 10 shows the PDF with the minimum Shannon entropy fault tolerant control by a 2D plot. Compared with Figure 7, it can be seen that with the rational entropy fault tolerant controller, the PDF before and after fault is more consistent, which means that the rational entropy fault-tolerant controller has better fault tolerance control effect.

## 6. Conclusions

In this paper, a collaborative fault tolerant control scheme is proposed for a class of collaborative systems. Firstly, a fault diagnosis observer is constructed in the first subsystem. This is followed by minimum rational entropy control based fault tolerant control scheme in the second subsystem and the third subsystem to make the uncertainty of the system output be minimized to compensate the influences caused by the fault. Finally, the proposed fault diagnosis and fault tolerant control algorithm has been examined by a simulation example. There are still many issues in this paper that are not comprehensive enough. The subsystem does not take into account the effects of the modeling error and the effects of delays which will be addressed in future studies. Only one way of collaboration mentioned in this paper, and there are many other types of collaboration in the actual system that needs further research. The number of subsystems in the collaborative system considered in this paper is small, and fault diagnosis and fault tolerant control of the collaborative system with more subsystems is also an interesting research direction.

## References

[1] Yao L.N., Lei C.H. (2017). Fault diagnosis and sliding mode fault tolerant control for non-Gaussian stochastic distribution control systems using T-S fuzzy model. Asian J. Control.

[2] Wang H. (2000). Bounded Dynamic Stochastic Systems: Modelling and Control.

[3] Bouallegue W., Bouslama S., Tagina M. (2017). Robust fault detection and isolation in bond graph modelled processes with Bayesian networks. Int. J. Comput. Appl. Technol..

[4] Zhou J.L., Li G.T., Wang H. (2014). Robust tracking controller design for non-Gaussian singular uncertainty stochastic distribution systems. Automatica.

[5] Yao L.N., Yin Z.Y., Wang H. (2014). Robust *H*_∞_ fault diagnosis for stochastic distribution systems with disturbance rejection performance. Int. J. Model. Ident. Control.

[6] Yao L.N., Qin J.F., Wang A.P., Wang H. (2013). Fault diagnosis and fault-tolerant control for non-Gaussian non-linear stochastic systems using a rational square-root approximation model. IET Control Theory Appl..

[7] Yao L.N., Guan Y.C., Wang A.P. (2015). Fault diagnosis and minimum entropy fault tolerant control for non-Gaussian singular stochastic distributions systems using square-root approximation. Int. J. Model. Ident. Contr..

[8] Maalej I., Abid D., Rekik C. (2017). State and fault estimation based on interval type-2 fuzzy inference system optimised by genetic algorithms. Int. J. Comput. Appl. Technol..

[9] Zhang J., Yue H., Zhou J.L. (2015). Predictive PDF control in shaping of molecular weight distribution based on a new modeling algorithm. J. Process Control.

[10] Sun X.B., Yue H., Wang H. (2006). Modelling and control of the flame temperature distribution using probability density function shaping. Trans. Inst. Meas. Control.

[11] Chen R.H., Mingori D.L., Speye J.L. (2003). Optimal stochastic fault detection filter. Automatica.

[12] Teng L., Wang Y.Y., Cai W.J., Li H. (2017). Robust model predictive control of discrete nonlinear systems with time delays and disturbances via T-S fuzzy approach. J. Process Control.

[13] Mayne D.Q., Seron M.M., Rakovic S.V. (2004). Robust model predictive control of constrained linear systems with bounded disturbances. Automatica.

[14] Yao L.N., Guan Y.C. Minimum entropy fault diagnosis and fault tolerant control for the non-Gaussian stochastic system. Proceedings of the 2016 American Control Conference (ACC).

[15] Li G., Zhao Q. (2017). Minimum entropy deconvolution optimized sinusoidal synthesis and its application to vibration based fault detection. J. Sound Vib..

[16] Yue H., Wang H. (2003). Minimum Entropy Control of Closed-Loop Tracking Errors for Dynamic Stochastic Systems. IEEE Trans. Automat. Control.

[17] Skaf Z., Wang H., Guo L. (2001). Fault tolerant control based on stochastic distribution via RBF neural networks. J. Syst. Eng. Electron..

[18] Qu Y., Li Z.M., Li E.C. (2012). Fault detection and diagnosis for non-Gaussian stochastic distribution systems with time delays via RBF neural networks. ISA Trans..

[19] Zakwan S., Ahamd B.A., Wang H. Fault detection and diagnosis for general discrete-time stochastic systems using output probability density estimation. Proceedings of the 50th IEEE Conference on Decision and Control and European Control Conference.

[20] Ren Y.W., Wang A.P., Wang H. (2015). Fault diagnosis and tolerant control for discrete stochastic distribution collaborative control systems. IEEE Trans. Syst. Man Cybern. Syst..

[21] Yao L.N., Wang H. A fault tolerant control scheme for collaborative two sub-systems. Proceedings of the 2005 IEEE International Symposium on, Mediterrean Conference on Control and Automation Intelligent Control.

[22] Ren Y.W., Wang H. Fault tolerant control for sequentially connected stochastic distribution continuous systems. Proceedings of the 2010 International Conference on Modelling, Identification and Control.

[23] Zhou J.L., Zhu H.J., Wang J. Minimum Entropy Control?. Proceedings of the 31th Chinese Control Conference.

[24] Yao L.N., Wang H. (2006). Fault diagnosis and tolerant Control for non-Gaussian stochastic distribution control Systems based on the rational square-root appriximation model. IET Control Theory Appl..

